
JOURNAL INFORMATION
==============================
NLM Title Abbreviation: Biospektrum (Heidelb)
Journal ID: Biospektrum (Heidelb)
NLM Journal ID: 9615685

Biospektrum

ISSN: 0947-0867
EISSN: 1868-6249

ARTICLE INFORMATION
==============================
PMCID: PMC8111374
DOI: 10.1007/s12268-021-1557-9
Article ID: 1557
Article version: 1
Subjects: Karriere, Köpfe & Konzepte

Buchrezension zu: The Doctor Who Fooled the World

Seifert Roland Seifert.Roland@mh-hannover.de

grid.10423.34 0000 0000 9529 9877 Medizinische Hochschule, Hannover, Deutschland
Electronic publication date: 2021 May 11
Print publication date: 2021
Volume: 27
Issue: 3
First page: 336
Last page: 336
Copyright: © Der Autor 2021
License: Dieser Artikel wird unter der Creative Commons Namensnennung 4.0 International Lizenz veröffentlicht, welche die Nutzung, Vervielfältigung, Bearbeitung, Verbreitung und Wiedergabe in jeglichem Medium und Format erlaubt, sofern Sie den/die ursprünglichen Autor(en) und die Quelle ordnungsgemäß nennen, einen Link zur Creative Commons Lizenz beifügen und angeben, ob Änderungen vorgenommen wurden. Die in diesem Artikel enthaltenen Bilder und sonstiges Drittmaterial unterliegen ebenfalls der genannten Creative Commons Lizenz, sofern sich aus der Abbildungslegende nichts anderes ergibt. Sofern das betreffende Material nicht unter der genannten Creative Commons Lizenz steht und die betreffende Handlung nicht nach gesetzlichen Vorschriften erlaubt ist, ist für die oben aufgeführten Weiterverwendungen des Materials die Einwilligung des jeweiligen Rechteinhabers einzuholen. Weitere Details zur Lizenz entnehmen Sie bitte der Lizenzinformation auf http://creativecommons.org/licenses/by/4.0/deed.de.
License URL: https://creativecommons.org/licenses/by/4.0/

issue-copyright-statement: © Springer-Verlag GmbH Deutschland, ein Teil von Springer Nature 2021

==============================

**The Doctor Who Fooled the World**
**Science, Deception and the War on Vaccines**
**Brian Deer**

*394 S., Johns Hopkins University Press, 2020. HC, 24,99 €.*
*ISBN: 9781421438009*
*Auch als E-Book erhältlich*

■ Ein aktuelleres Buch kann es nicht geben: Der britische Investigativjournalist Brian Deer zeichnet in diesem Buch einen der größten Wissenschaftsskandale der jüngsten Vergangenheit nach.

Normalerweise beschäftigen Wissenschaftsskandale nur die Spezialisten, aber leider hatte dieser Skandal bis heute weitreichende gesellschaftliche Folgen: Andrew Wakefield, ein britischer Arzt, dem inzwischen die Berufserlaubnis entzogen wurde, setzte vor ca. 15 Jahren die Behauptung in die Welt, dass Impfstoffe eine besondere Form von kindlichem Autismus verursachen würden. Dies führte dazu, dass die Impfbereitschaft in etlichen Ländern herunterging und es wieder Ausbrüche von längst verschwunden geglaubten Infektionserkrankungen wie Masern gab – mit etlichen (vermeidbaren) Todesfällen. Obwohl Wakefield des Betrugs überführt wurde und die entsprechenden Publikationen in sehr namhaften Journalen zurückgezogen wurden, hält sich hartnäckig das Gerücht, Impfungen verursachten Autismus, und wird in entsprechenden Publikationen von Impfgegnern wie „Virus-Wahn“ von T. Engelbrecht und C. Köhnlein unkritisch wiederholt. Impfgegnerschaft stellt gerade bei der Bewältigung der SARS-CoV-2-Pandemie ein immenses Problem dar. Nach einem furiosen Impfstart hat Israel jetzt ein Problem, und wer weiß, wie es in Deutschland laufen wird.

Vor diesem Hintergrund ist das Buch über den Wakefield-Skandal sehr nützlich, um zu verstehen, wie es überhaupt so weit kommen konnte und warum eindeutig als Fälschung identifizierte Publikationen in der Öffentlichkeit eine so lange Halbwertszeit besitzen. Leider gestaltete sich die Lektüre des Buchs anstrengender als gedacht. Der Schreibstil des Autors ist sehr speziell und für einen Wissenschaftler, der sachliche trockene Texte gewöhnt ist, nicht leicht verdaubar. Außerdem verliert sich der Autor manchmal sehr in unwichtigen Details. Dennoch gebührt Brian Deer großer Dank dafür, diesen Skandal exponiert und aufgeklärt zu haben. Denn Wakefield war kein Einzeltäter, sondern konnte seine Fälschungen nur mithilfe von Kollegen, auf *impact* bedachten ehrgeizigen Editoren wissenschaftlicher Zeitschriften und potenten Geldgebern aus dem Dunstkreis von Impfgegnern verbreiten.

Der Rezensent empfiehlt dem Leser, zunächst die *timeline* im Appendix des Buchs zu lesen, aus der die Chronologie der Ereignisse gut hervorgeht. Dann verliert man sich nicht so sehr in den einzelnen Kapiteln. Insgesamt war das Buch sehr viel weniger Lesegenuss und weniger spannend als erwartet, was nicht am Inhalt liegt, sondern am Schreibstil. Aber das Preis-Leistungs-Verhältnis stimmt, und man hat mit diesem Buch ein aktuell passendes Geschenk für jeden Wissenschaftlerkollegen parat.

Ein weiteres Buch, das mit dem hier rezensierten Buch große thematische Überlappungen aufweist, ist „Stuck: How Vaccine Rumors Start -- And Why They Don't Go Away“ von Heidi J. Larson. Letzteres Buch betont vor allem die unrühmliche Rolle sogenannter „sozialer“ (in diesem Falle aber asozialer) Medien bei der Verbreitung von Lügen und Verschwörungstherorien. ■

*Roland Seifert,*
*Medizinische Hochschule Hannover,*
*Seifert.Roland@mh-hannover.de*

Diese Rezension erscheint **Open Access**.*

* **Funding note:** Open Access funding enabled and organized by Projekt DEAL.
**Open Access:** Dieser Artikel wird unter der Creative Commons Namensnennung 4.0 International Lizenz veröffentlicht, welche die Nutzung, Vervielfältigung, Bearbeitung, Verbreitung und Wiedergabe in jeglichem Medium und Format erlaubt, sofern Sie den/die ursprünglichen Autor(en) und die Quelle ordnungsgemäß nennen, einen Link zur Creative Commons Lizenz beifügen und angeben, ob Änderungen vorgenommen wurden. Die in diesem Artikel enthaltenen Bilder und sonstiges Drittmaterial unterliegen ebenfalls der genannten Creative Commons Lizenz, sofern sich aus der Abbildungslegende nichts anderes ergibt. Sofern das betreffende Material nicht unter der genannten Creative Commons Lizenz steht und die betreffende Handlung nicht nach gesetzlichen Vorschriften erlaubt ist, ist für die oben aufgeführten Weiterverwendungen des Materials die Einwilligung des jeweiligen Rechteinhabers einzuholen. Weitere Details zur Lizenz entnehmen Sie bitte der Lizenzinformation auf http://creativecommons.org/licenses/by/4.0/deed.de.

Funding note

Open Access funding enabled and organized by Projekt DEAL.

